# Evaluation of the effect of HIV virus on the digestive flora of infected versus non infected infants

**DOI:** 10.11604/pamj.2019.34.24.15039

**Published:** 2019-09-11

**Authors:** Celine Nguefeu Nkenfou, William Baiye Abange, Hortense Kamga Gonsu, Nelly Kamgaing, Emilia Mbamyah Lyonga, Jean de Dieu Anoubissi, Alexis Ndjolo, Paul Koki

**Affiliations:** 1 Chantal Biya’s International Reference Centre (CBIRC), Centre for HIV/AIDS Prevention and Management, Yaounde, Cameroon; 2 Department of Biological Sciences, Higher Teachers Training College, University of Yaounde I, Cameroon; 3 University Teaching Hospital, Yaounde, Cameroon; 4 Catholic University of Central Africa, Yaounde, Cameroon; 5 Faculty of Medicine and Biomedical Sciences, University of Yaounde I, Yaounde, Cameroon; 6 National AIDS Control Committee, Ministry of Public Health, Yaounde, Cameroon; 7 Centre Mère Enfant, Fondation Chantal Biya, Yaounde, Cameroon

**Keywords:** HIV, digestive flora, infants, opportunistic/pathogenic bacteria

## Abstract

**Introduction:**

HIV infection is characterized by changes in the composition and functions of gut microbiota. We carried out a study aiming at comparing the compositional changes of the digestive flora of HIV infected infants versus that of non infected infants in Cameroon.

**Methods:**

A case-control study was carried out during which stool sample was collected from each participant after obtaining the proxy consent. Stools were cultured using aerobic, strict anaerobic, 10% CO2 and micro-aerophilic conditions and specific culture media and bacteria were identified biochemically. Fisher's exact test was used for data analyses.

**Results:**

From the 80 infants enrolled for the study, 33 (41.3%) were HIV positive. A statistically significant difference was observed between the number of infected versus non infected infants harboring the following bacteria: *Clostridium* spp. (P=0.009); *Enterococcus* spp. (p<0.001); *Klebsiella* (p<0.001); *Shigella* (<0.001); *Staphylococcus aureus* (p=0.006) and *Streptococcus* spp. (P=0.015). Among infected infants, WHO-stage 3 and 4 infants harbored more opportunistic bacteria than stage 1 and stage 2 and *Bacteriodes* spp. population was depleted as the disease progresses, although not significantly. There was an imbalance in bacteria flora in HIV infected infants harboring qualitatively more bacteria including more opportunistic and pathogenic bacteria than in HIV non-infected infants.

**Conclusion:**

HIV infected infants presented a qualitatively different flora from HIV non infected infants. They habored more pathogenic bacteria Than non infected infants. Systematic stool culture could benefit for follow-up of HIV infected infants to reduce the risk of gastrointestinal disorders and thus the risk of high morbidity or high mortability.

## Introduction

Since the first cases of human immunodeficiency virus (HIV) infection were identified in 1981, the number of infants infected with HIV has increased dramatically in developing countries because the number of HIV-infected women of childbearing age has risen [1]. However, great advances have been made in developed countries to control transmission of the virus from mother to infant. In developing countries some progresses have also been made. Vertically transmitted HIV can cause rapidly progressive, chronically progressive, or adult like disease in which a significant clinical latency period occurs before symptoms appear. The disease progression depends on several factors including the frequency of bacterial infections. Microbial translocation (MT) from the gut has been implicated in driving immune activation, increasing morbidity and mortality in HIV [2]. On the other hand, intestinal flora plays an important role in the defense mechanism in human beings [3]. Specifically, bacterial flora has been shown to play a beneficial role when in equilibrium, but becomes harmful when there is imbalance. While the general dogma is that the placental barrier keeps infants sterile throughout pregnancy, increasing evidence suggests that an infant´s initial bacterial inoculum can be provided by its mother before birth [4, 5] and is supplemented by maternal microbes during the delivery [6] and breastfeeding [7, 8] processes. Soon after birth, infant flora evolves depending on several factors like mode of delivery, nutrition, age and environment [3, 9]. Fooks *et al.* (2007) [10] reported that the bacterial population in the intestine is quantitatively and qualitatively balanced; each genus has its own growth niche maintaining an "optimal balance" for the physiological performance of the digestive system. However, numerous factors can alter it, either by stimulating beneficial actions through an increase in the number of certain bacteria (*Bifidobacteria* and *Lactobacilli*), or by stimulating the proliferation of bacteria considered pathogenic (*Clostridia*, certain species of *Bacteroids*, etc.). Knowing that 60% of CD4+ T cells in the human body reside in gut-associated lymphoid tissue, and that the reconstitution of these cell populations and the gut microbial composition is incomplete even under HAART, the human intestine has recently become the focus of attention in HIV research [11]. Moreover, changes in gut microbial composition and function in HIV-positive individuals, aside from being secondary to HIV infection, may also play direct roles in mediating some disease manifestations (recurrent bacterial infections) [11]. The intestinal flora, if well characterized could be balanced before the onset of infection using probiotics. It is for this reason that this research project was initiated with the aim to examine the digestive flora of HIV infected infants born to HIV sero-positive mothers in comparison to that of HIV non-infected infants.

## Methods

**Study area and population:** this study was conducted in Yaounde, Centre Region, the nation's capital city that attract people from other parts of the country and globally for work, education or leisure. Participants were selected based on whether they met the inclusion criteria. HIV patients irrespective of their gender and age were eligible to participate. Excluded from the study were individuals who were on any other antibiotic medication other than prophylatic co-trimoxazole. Participants were randomly recruited into the study from two treatment centers until a convenient sample size was attained. We informed the parents of the participating children of the nature and objectives of the study, and their right of refusal to participate in the study or to withdraw at any time, without jeopardizing their right of access to other health services. After parental consent, fresh stool samples were collected in dry sterile stool container, registered and transported to the Bacteriology Laboratory of Yaounde University Teaching Hospital for analyses using standard operating procedures.

**Study design and period:** this was a cross-sectional study. Briefly, the cases were HIV infected children and the control group were HIV non-infected children aged between 3 months to 24 months. They were enrolled in two HIV treatment centers: Center for Mother and Child, Chantal Biya's Foundation Yaounde and the Pediatric Service-Yaounde of the University Teaching Hospital (YUTH). Controls were enrolled from the Melen neighborhood from April to October 2013.

**Sampling technique:** a time limited sampling technique was used, where patients were consecutively recruited into the study. Participants were enrolled into the study provided their parents/guardians gave their consent and met the inclusion criteria. Our sample size was a convenient one since it was a pilot study.

**Process of the survey and sample collection:** HIV infected children consulting at the study sites on their normal appointment day or for an emergency problem were requested to participate in the study. After obtaining parental informed consent, a questionnaire was administered to each participant's parent by a nurse specifically trained for this task. The participants were instructed on how to produce stool specimen avoiding contamination. Fresh stool sample was collected in a dry sterile stool container. Two ml of blood were collected from each infant to confirm their HIV status. For infants aged less than 18 months, HIV DNA PCR (Amplicor HIV-1DNA test, Roche Diagnostics, Branchburg, NJ) was used. For those aged more than 18 months, the serology test (Determine HIV1/2 test (Alere, Chiba, 270-2214, Japan) coupled to Oraquick test (OraSure Technologies, Thailand)) was done. The fresh stools were transported to the laboratory within 30 minutes for culture. We ensured none of the children participating was taking antibiotic medications at the time of recruitment other the prophylatic co-trimoxazole.

**Laboratory procedures and stool analysis:** using standard operating procedures at the Medical Microbiology/Bacteriology Laboratory of the YUTH, we performed macroscopic examination of the fresh stool samples to note and appreciate the stool consistency, color, odor and lastly observed for the presence of blood or mucus. The fresh stools were then inoculated for the identification of bacteria using specific growth media (Manitol salt agar, MacConkey agar, Blood agar, Shaedler Anaerobe blood agar plus antibiotic inhibitors Vancomycine and Kanamycine, Campybrilliance agar, Reinforced Clostridium differential broth, MRS agar, Bile Aesculin agar. After inoculation, we then incubated at different conditions; aerobic, strict anaerobic and micro-aerophilic conditions. Biochemical tests for microorganism identification was done using conventional classical gallery (oxidase and catalase, urea-indole, Simon citrate fermentation, gas production, lactose and glucose fermentation) and BioMérieux´s API (5 rue des Aqueducs 69290 Craponne, France) commercial identification kits for Gram positive and Gram negative bacteria. For microscopic examination, we performed direct Gram stain to determine, quantify and appreciate the bacteria flora. It should be noted that we performed the culture before microscopic examination to avoid contamination.

**Data collection:** data were collected using a designed questionnaire. Information such as: age, sex, mode of delivery, feeding mode and HIV status were collected. For each participant, their medical records were used to get information concerning their HIV status if available and clinical stage according to the WHO classification. Biological data collected after biochemical characterizations were entered into the database.

**Statistical analysis:** the data collected from the questionnaires and biological analyses were entered in CSPRO version 20.0, for analyses. Statistics performed included the chi-square test for group comparison. P-value less than 0.05 was considered as statistically significant at 95% confidence interval (CI) according to Fisher's exact test.

**Administrative and ethical considerations:** administrative authorizations were obtained from the UTHY and CMC/CBF. Ethical clearance was obtained from the National Ethical Committee, authorization n^o^. 2013/02/N^o^026/L/CNERSH/SP. The enrolled infants were those whose parents had signed the proxy consent form. All the participating parents were assured of confidentiality, guaranteed by the attribution of a codified number.

## Results

**Description of study population:** our population constituted of 80 infants of age between 3 months and 24 months. The majority of the infants recruited during the study were male 49 (61.25%), the rest, 31 (38.75%) were female. From these, we had 33 (41.2%) HIV positive, 15 (18.8%) exposed to HIV but not infected and 32 (40%) negatives (not exposed to HIV). Thirty nine were exclusively breastfed, 24 were under mixed feeding and 17 infants were on exclusive artificial feeding. According to the delivery mode, 66 (82.5%) were born by normal route of which 30 were HIV positive (45%) and 14 (17.5%) were born by caesarean; of which 3 were HIV positive (21%). Thirty two of the HIV infected infants were on ARV treatment (97%). Of the 80 infants, 9 were on antibiotics (4 HIV negative and 5 HIV positive); these infants were excluded from the analyses. Five of the 80 infants had diarrhoea (one HIV negative and 4 HIV positive) and were excluded from the analyses. According to the WHO classification (WHO/HIV/2005.02), of the 33 HIV infected infants, 15 (45.45%) were at stage 4, 11 (33.33%) at stage 3, one (1.25%) at stage 2, and 6 (18.18%) infants were at stage 1. These data are presented in Table 1.

**Table 1:** Socio-demographic presentation of the study population

Characteristic	N (%)
Mean age	15±7
**Sex**	
Male	49(61.3)
Female	31(38.8)
**HIV status**	
Positive	33(41.3)
Negative (exposed un infected + non-exposed)	47(58.7)
**Feeding mode**	
Mother breast milk	39 (48.7)
Artificial milk	17(21.3)
Mixed feeding	24(30.0)
**Delivery mode**	
Normal route	66(82.5)
Caesarean	14(17.5)
**Mother education level**	
Illiterate	3(3.8)
*Primary*	17(21.3)
Secondary	38(47.5)
Higher	22(27.5)
**On Antibiotic**	
Yes	9 (11.3)
No	71 (88.7)

**Bacteria isolation:** seventeen bacteria species were isolated overall in our study population. *Lactobacillus spp.* (91.3%) *Bifidobacterium* (81.3%), *Campylobacterium* (67.5%), *Escherichia coli* (87.5%) and *Streptococcus spp.* (70%) were the most abundant. Bacteriodes were identified from only 32% of infants. Pathogenic as well as opportunistic bacteria were also isolated (*Clostridium spp.* (35%), *Staphylococcus aureus* (6.3%), *Klebsiella spp.* (6.3%), Shigella spp. (11.3%).

**Comparison of bacteria isolated according to the HIV status:** among the HIV infected children, *Lactobacillus spp.* (96.97%), *Streptococcus spp.* (84.85%) and *Bifidobacterium spp.* (81.81%) were mostly identified. Whereas in HIV negative infants, high frequency of E. coli (93.62%), *Lactobacillus spp.* (87.23%) and *Bifidobacterium spp.* (80.85%) was observed. From HIV infected infants, high diversity (15 different types of bacteria species) was observed with 6 out of 15 being opportunistic and pathogenic bacteria in immune-compromised patients: *Shigella spp.* (24.24%), *Staphylococcus aureus* (15.15%), *Klebsiella spp.* (12.12%), *Pseudomonas spp* (3.03%), *Acinetobacter spp.* (3.03%) and *Proteus spp.* (3.03%). We observed the absence of *Actinomycetes* and *Enterobacter spp.* from HIV positive infants and the absence of *Acinetobacter spp. Proteus spp*. and *Staphylococcus aureus* from HIV negative infants. Statistically we found out that opportunistic bacteria (*Shigella spp.* p<0.001), *Klebsiella spp.* (p<0.001), and *Staphylococcus aureus* (p=0.006) were associated with HIV status; as well *Clostridium spp.* (p=0.009), *Enterococcus spp.* (<0.001) and *Streptococcus spp*. (p=0.015) were more frequent in HIV infected infants. There was no statistical difference in the number of infected versus non infected infants harboring *Lactobacillus spp. Bifidobacterium spp* and *Staphylococcus spp.* These data are presented in Table 2. We also observed that many HIV stage 4 infants harbored more opportunistic bacteria than infants of other HIV stages; *Acinetobacter spp, Klebsiella spp. Proteus spp. Shigella spp* and *Staphylococcus aureus*.

**Table 2:** Comparison of selected bacteriological profiles by HIV status

Bacteria isolated	HIV status of children		P value
	Positive n(%) N=33	Negative n(%) N=47	
Gram Negative Anaerobic strict bacilli (*Bacteroides spp.*)	11(33.3)	15(32)	0.894
*Bifidobacterium spp.*	27 (82)	38(81)	0.913
*Campylobacter spp.*	25(75.8)	29(61.7)	0.186
*Clostridium spp.*	17(51.5)	11(23.4)	0.009
*Enterococcus spp.*	17(52)	6(12.8)	<0.001
Enterobacteriaceae	30(91)	46(98)	0.159
*Klebsiella*	4(12.1)	1(2.1)	<0.001
*Lactobacillus spp.*	32(97)	41(87.2)	0.129
*Shigella*	8(24.2)	1(2.1)	<0.001
*Staphylococcus aureus*	5(15.2)	0(0)	0.006
*Staphylococcus spp.*	11(33.3)	13(27.6)	0.586
*Streptococcus spp.*	28(84.9)	28(59.6)	0.015

**Exposure to HIV:** we did not observe any difference in the digestive flora of HIV exposed and non-infected infants versus non-exposed HIV negative infants.

**Analyses according to the delivery mode:** according to the delivery mode, we isolated more bacteria species from infants born by vaginal route (17 species) than from infants born by caesarean section (8 species). The difference in the frequency of *Staphylococcus spp*. between infant delivered through the vaginal route and caesarean was statistically significant (p= 0.002, 64.3% versus 22.7%).

**Analyses according to the feeding options:** according to the feeding mode, our study showed that there was no statistically significant difference in the bacteria isolated except for *Staphylococcus spp*. which was mainly harbored by breastfed infants (88.9%) compared to infants on mixed feeding (11.1%), p=0.005. *Bifidobacterium* were slightly more frequently isolated from infants on breastfeeding (67.3% versus 32.7% in mixedfed infants) (p=0.059). The difference in *Lactobacillus spp*. isolated was statistically significant (found mostly from mixedfed infants (64.9%) compared to 35.1% in artificially fed infants) with p=0.024. Likewise, *Staphylococcus spp.* were mainly isolated from artificially fed infants (75% compared to 25% for mixed fed infants) p=0.041.

## Discussion

Our work aimed at evaluating the effect of HIV infection on the digestive flora of HIV infected infants using biochemical methods for bacteria identification. From this study 80 participants were enrolled from the CMC/CBF, the UTHY and the community. This was a case-control study where 33 (41.25%) HIV positive infants and 47 (58.75%) HIV negative infants were enrolled. 32 of the HIV infected infants were on ARV treatment while only one was still waiting initiation which should tally with the WHO 2010 recommendations: "Start ART immediately upon diagnosis." Unfortunately most of the HIV positive infants (78.78%) were already at Stage 3-4 based on the WHO classification. This may imply that they were initiated on ART treatment very late; or that they were initiated with wrong drugs due to the acquisition of drug resistant strains from their mothers. We looked at bacteria shed in the stools, which is an easy to collect biological material. Bacteria make up most of the flora in the colon and up to 60% of the dry mass of feces [12, 13]. An estimate of 300 to 1000 different species live in the gut [14, 15], with most estimated at about 500 [16-18]. However, it is probable that 99% of the bacteria come from about 30 or 40 species [12]. According to the delivery mode, we isolated more bacteria species from infants born by vaginal route than from infants born by caesarean section. The difference in the frequency of *Staphylococcus spp*. between infant delivered through the vaginal route and caesarean was statistically significant (p= 0.002, 64.3% versus 22.7%). A number of studies have shown a relationship between the mode of delivery and the colonization of the infant intestine [19-24]. According to the feeding mode, our study showed a differential frequency in harboring *Staphylococcus spp. Bifidobacterium* and *Lactobacillus spp*. This is in agreement with other studies that showed a relationship between the digestive flora and the mode of nutrition [9, 19, 25, 26]. We will like to clarify here that formula feeding in Cameroon is based on two main types: plain and enriched. In Cameroon, pediatric milks (0-6 months) are enriched with probiotics (*Lactobacillus* or Bifidobacteria) and prebiotics (mainly the fructo-oligosaccharides and the galacto-oligosaccharides). These milks represent more than 50% on the market.

The diversity of bacteria identified was analysed according to the HIV status of the infants. Qualitatively we isolated more bacteria in HIV positive children (15 different species of bacteria) than in exposed HIV negative infants and non-exposed negative (11 different species of bacteria). Statistically, *Clostridium spp. Enterococcus spp. Stapylococcus aureus* and *Streptococcus spp*. were significantly associated with HIV positive status (p=0.009, P=0, P=0.006, P=0.015 respectively). The number of bacteria isolated increases from stage 1 to stage 4 (from 11 to15). HIV infection negates both gut bacteria and epithelial barriers, then propagate the infection [27]. There was an observed shift in gut composition from protective species to pro-inflammatory disease-inducing bacterial species that promote viral replication and chronic immune activation. *Bacteroides* species also known to benefit their host by excluding potential pathogens from colonizing the gut were least frequently isolated from infected infants. This dysbiosis observed in infants has also been observed in adults [28-30]. Five (15.15%) of the 33 HIV infected infants were on antibiotics and 4 (8.51%) of 47 HIV negative were also on antibiotics with no significant difference in bacteria flora. Normally, all the HIV infected infants were supposed to be on cotrimoxazole until 5 years old according to WHO Guidelines on cotrimoxazole prophylaxis for HIV-related infections among infants, adolescents and adults: recommendations for a public health approach. Among the HIV infected children, *Lactobacillus spp*. (96.97%), Streptococcus spp. (84.85%) and Bifidobacterium spp. (81.81%) were mostly identified. Whereas in HIV negative infants, high frequency of E. coli (93.62%), *Lactobacillus spp.* (87.23%) and *Bifidobacterium spp.* (80.85%) was observed. In a study done on younger infants (few days of life), the main bacterial groups found in HIV negative infant's faeces were Bifidobacterium, Streptococci and Enterococci [31]. This may be due to age, feeding option and environment.

In our study population, opportunistic bacteria (*Shigella spp. Klebsiella spp. Proteus spp. Pseudomonas spp.* and *Staphylococcus aureus*) were significantly associated with HIV positive infants. Their immune-compromised state promotes the proliferation of these opportunistic bacteria. Bacterial infections are the most common opportunistic infections in HIV. Since the advent of highly active antiretroviral therapy (HAART), the incidence of these infections is on the decline. However, globally there is a significant lack of access to care among HIV patients because of limited drug availability especially pediatric formulations. Furthermore, non-compliance and drug resistance can hinder viral suppression, predisposing patients to opportunistic infections. In other not to bias the result obtained from our study we mostly enrolled infants who presented at the hospital for their routine follow-up (less or no clinical signs and symptoms). This is why we had 11.25% on antibiotics, 6.25% with diarrhea and 3.75% with constipation; which were excluded from our analyses. So our study was limited by lack of possibility to cover all the bacteria living in the GIT, and thus was confined to those that could be cultured. Not all the species in the gut have been identified because most cannot be cultured [15], and making identification difficult. An effort to better describe the microflora of the gut and other body fluids using newly developed non-culture based technologies [32] has been achieved. A project termed the "Human Microbiome Project" [33] has been initiated and takes advantage of new, high-throughput technologies to characterize the human microbiome.

## Conclusion

We aimed at looking at the effect of HIV infection on the digestive flora of HIV infected infants. Our results showed that HIV infected infants harbor qualitatively more bacteria than HIV non-infected infants. We found that, as HIV disease progresses the digestive flora changes qualitatively and also that as the disease advances (Stage 1-4) the number of opportunistic bacteria also increases. HIV infected infants harbor more opportunistic bacteria than non-infected infants. The delivery mode affected the flora of HIV infected infants compared to non-infected infants (*Staphylococcus spp*.). As well, the feeding mode affected the flora (*Staphylococcus spp.* and *Lactobacillus*). This is the first study to our knowledge done on the flora of HIV positive versus negative infants in Cameroon. Data obtained highlights that bacteria present in infants' gastrointestinal tract are important parameters in the progression of HIV/AIDS disease. Targeting the balance of digestive flora is thus crucial for the management and effective treatment of HIV co-morbidities. For limited resources countries, stool culture could be used for better management of HIV infected infants in order to reduce the gut disorders.

### What is known about this topic

- Studies have further shown that HIV-infected adults experience dysbiosis in their microbiome;
- Culture-based and molecular methods looking at the microbiome of mother (breast milk) and infant (≤14 days of life-gut) pairs have shown some associations with HIV infection in southern Mozambique. The main bacterial groups found in infant's (uninfected) faeces were Bifidobacterium, Streptococci and Enterococci;
- Maternal HIV infection Influences the Microbiome of HIV-exposed uninfected infants.

### What this study adds

- Differential digestive flora of HIV infected and uninfected infants aged 3-24 months using culture-based methods;
- Higher diversity bacteria in HIV infected compared to uninfected infants.

## Competing interests

The authors declare no competing interests.
